# Aerosol Dispersion During Mastoidectomy and Custom Mitigation Strategies for Otologic Surgery in the COVID-19 Era

**DOI:** 10.1177/0194599820941835

**Published:** 2020-07-14

**Authors:** Divya A. Chari, Alan D. Workman, Jenny X. Chen, David H. Jung, Dunia Abdul-Aziz, Elliott D. Kozin, Aaron K. Remenschneider, Daniel J. Lee, D. Bradley Welling, Benjamin S. Bleier, Alicia M. Quesnel

**Affiliations:** 1 Department of Otolaryngology–Head and Neck Surgery, Massachusetts Eye and Ear, Harvard Medical School, Boston, Massachusetts, USA

**Keywords:** mastoidectomy, COVID-19, severe acute respiratory syndrome coronavirus-2, SARS-CoV-2, virus transmission, aerosol, aerosol generating procedure, aerosolization, airborne, otology, neurotology, barrier drape, personal protective equipment, health care providers, safety, OtoTent

## Abstract

**Objective:**

To investigate small-particle aerosolization from mastoidectomy relevant to potential viral transmission and to test source-control mitigation strategies.

**Study Design:**

Cadaveric simulation.

**Setting:**

Surgical simulation laboratory.

**Methods:**

An optical particle size spectrometer was used to quantify 1- to 10-µm aerosols 30 cm from mastoid cortex drilling. Two barrier drapes were evaluated: OtoTent1, a drape sheet affixed to the microscope; OtoTent2, a custom-structured drape that enclosed the surgical field with specialized ports.

**Results:**

Mastoid drilling without a barrier drape, with or without an aerosol-scavenging second suction, generated large amounts of 1- to 10-µm particulate. Drilling under OtoTent1 generated a high density of particles when compared with baseline environmental levels (*P* < .001, *U* = 107). By contrast, when drilling was conducted under OtoTent2, mean particle density remained at baseline. Adding a second suction inside OtoTent1 or OtoTent2 kept particle density at baseline levels. Significant aerosols were released upon removal of OtoTent1 or OtoTent2 despite a 60-second pause before drape removal after drilling (*P* < .001, *U* = 0, n = 10, 12; *P* < .001, *U* = 2, n = 12, 12, respectively). However, particle density did not increase above baseline when a second suction and a pause before removal were both employed.

**Conclusions:**

Mastoidectomy without a barrier, even when a second suction was added, generated substantial 1- to 10-µm aerosols. During drilling, large amounts of aerosols above baseline levels were detected with OtoTent1 but not OtoTent2. For both drapes, a second suction was an effective mitigation strategy during drilling. Last, the combination of a second suction and a pause before removal prevented aerosol escape during the removal of either drape.

During the acute phase of the COVID-19 pandemic, major disruptions occurred in the health care sector.^1^ Clinicians require strategies to safely practice medicine, particularly in the setting of persistent shortages of widely available testing^2^ and personal protective equipment (PPE)^3^ and a lack of contact tracing in the community, as attempted in other countries.^4,5^

Otolaryngologists may be at increased risk for occupational exposure, as studies show that the use of a high-powered drill is associated with aerosol generation.^6-10^ The Centers for Disease Control and Prevention and the World Health Organization have recommended higher levels of PPE for aerosol-generating procedures.^11,12^ Local source control may be an effective adjunctive strategy to mitigate viral transmission risk; however, there are currently no standardized local source-control strategies for otologic surgery. In a recent study, we illustrated the plume of aerosolized debris generated by mastoidectomy, quantified particulate (≥100 µm) dispersion in a 360° field around the surgical site, and demonstrated the effectiveness of a simple barrier drape attached to the microscope (previously termed “OtoTent” and referred to as “OtoTent1” in this study) for reducing large-particulate dispersion.^6^

Herein, we investigate the generation of aerosols during mastoidectomy in human cadaveric specimens for droplets and particulates sized 1 to 10 µm, which includes the range commonly associated with airborne disease spread.^13^ Furthermore, we evaluate the efficacy of 2 barrier drapes to decrease exposure to these aerosols, including OtoTent1 and a novel prototype customized for otologic surgery, OtoTent2. Additionally, we evaluate the effect of adding a second suction (SS) to the field, with or without barrier drapes.

## Methods

### Preparation of Specimens and Surgical Simulation

The protocol was deemed exempt by the Massachusetts General Brigham Institutional Review Board (protocol 2020P001151). Surgical simulation was performed on 6 ears from 3 thawed fresh-frozen cadaveric head specimens. All experiments were performed in a surgical laboratory set at 72 °F and equipped with air exchangers operating at a rate of 6 air changes in the room per hour. A right-handed surgeon completed all surgical conditions. The surgeon performed a cortical mastoidectomy and drilled for 1 minute for each condition. The microscope was a wall-mounted Zeiss OPMI Pico (Carl Zeiss; Meditec AG) with an objective lens focal distance of 250 mm. An otologic drill (Midas Rex Legend Stylus) with a compatible 6-mm round fluted bur (Xomed) and 5-mm diamond bur (Medtronic, Inc) was used at 70,000 rpm for drilling. The otologic drill had an attached irrigation port set to 10 mL/min. In all conditions, a 12-Fr suction was used in the surgeon’s nondominant hand, with the suction tip maintained approximately 1 cm from the drill bur. The 12-Fr suction was connected to wall suction at a pressure of 538 mm Hg (measured by a digital pressure gauge; Cole-Parmer) and resulted in an air flow rate of 32 L/min (measured by a variable area flowmeter; Cole-Parmer).

### Aerosol Sampling

**Figure 1.**
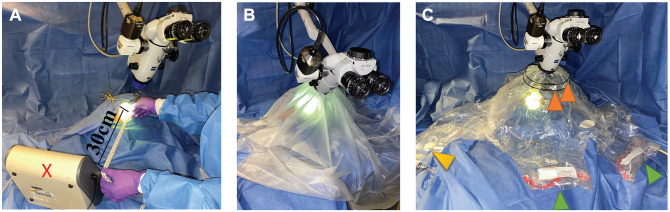
Experimental setup. (A) No barrier. Optical particle sizer (red ×): 30 cm from surgical field. (B) OtoTent1. (C) OtoTent2. Arm ports, green arrowheads; instruments/suction ports, yellow arrowhead; collapsible frame, orange arrowheads.

An optical particle sizer (OPS 3330; TSI Inc) placed 30 cm from the ear canal (**Figure 1A**) measured particle number and size distribution in the range of 1 to 10 µm, with a flow rate of 1.0 L/min through a 3-mm port. Total particle counts by size were collected in 10-second intervals for the duration of each experiment, with replicates performed for each test condition. Background measurements were taken before each experiment for 60 seconds, and experiments proceeded only if the aerosol concentration was at baseline.

### Barrier Drapes

Two types of barrier drapes were fashioned. OtoTent1 was created with a 1060 Steri-Drape (3M), affixed around the microscope lens after a 6-cm hole was cut into the drape, enclosing the cadaveric head specimen (**Figure 1B**) as previously described.^6^ OtoTent1 was draped over the surgical field and secured in 3 cardinal locations. The surgeon’s hands and instruments were passed under the drape to access the surgical field.

**Figure 2.**
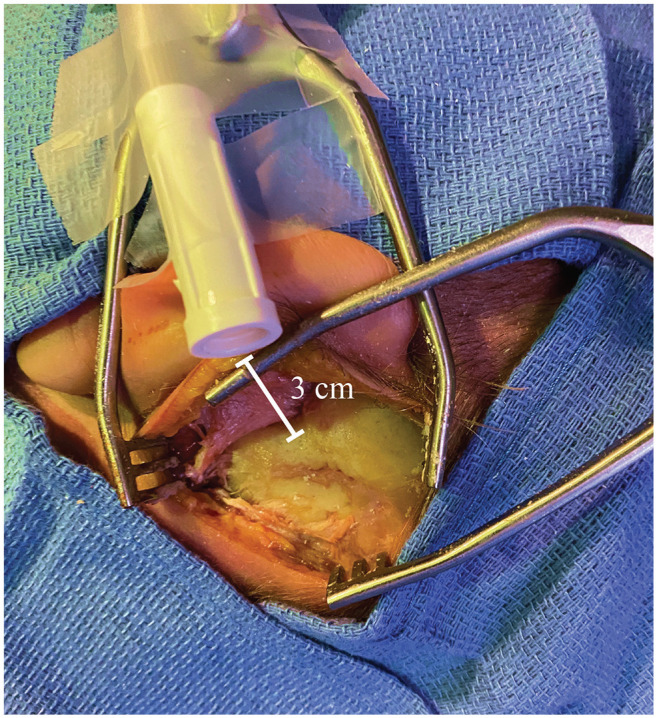
Experimental setup of second suction. Suction tubing was attached to the cadaver 3 cm from the mastoid cortex to continuously evacuate particles.

OtoTent2 was a custom prototype based on a modified Zeiss OPMI microscope drape (Carl Zeiss, Meditec AG; **Figure 1C**) created by Grace Medical. It was attached to the outer perimeter of the microscope lens with a 9-cm opening and secured with an elastic cinch cord. OtoTent2 contained 2 arm ports to accommodate the surgeon’s hands, with reinforced stiffened entry points to facilitate arm placement. The arm ports were not sealed around the surgeon’s arms. A third port accommodated the suction and otologic drill, sealed circumferentially with a piece of Velcro. OtoTent2 created a 3-dimensional enclosed space with a plastic drape that formed the “floor.” A 12-cm-diameter hole was cut into the floor and loosely adhered (but not sealed) to the cadaveric head around the surgical site. Neither OtoTent1 nor OtoTent2 was a sealed system, and potential sources of air leak are illustrated in **Figure 2**. Volumes for OtoTent1 and OtoTent2 were based on a truncated cone shape and pyramidal shape, respectively, and found to be 40 L and 37 L, respectively.

### SS Setup

**Figure 3.**
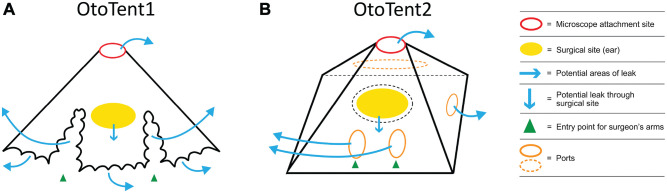
Barrier drape schematic. (A) OtoTent1. No floor: surgeon’s hands and instruments pass under the drape. (B) OtoTent2. Specialized drape with floor: arm ports for surgeon’s hands and port for instruments/suction.

Where indicated, the open end of an SS tubing (3/16″× 6′; Cardinal Health) was secured 3 cm from the mastoid cortex to continuously scavenge aerosolized particles from the air near the surgical site (**Figure 3**). (Of note, it was not used to suction liquid runoff.) The SS was connected to a second wall suction (separate from that with the 12-Fr suction), with a measured air flow rate of 65 L/min. The noise level from the SS was measured with a sound level meter (Decibel X; SkyPaw Co, Ltd) and found to be 53 dB. In contrast, the noise level of the 12-Fr suction was 73 dB.

### Test Conditions

A cortical mastoidectomy was performed under the microscope (with no barrier drape) with drilling for 1 minute. All procedures were performed with a 6-mm round fluted (“cutting”) otologic bur. To assess the 2 barrier drapes, the following conditions were tested with simulated cortical mastoidectomy: no barrier drape, OtoTent1, and OtoTent2 (**Figure 1**). Each condition was tested with and without the use of an SS fixed in the surgical field to continuously evacuate particles. The SS was turned on at the start of drilling and left on during barrier removal and subsequent particulate measurements. The drape was removed either immediately upon cessation of drilling or after a 60-second rest period. The surgeon’s arms were removed from the field at the conclusion of drilling regardless of whether the drape was removed immediately or after a delay.

### Statistical Analysis

Stata (v 13; StataCorp) software was used for statistical analysis to assess differences in airborne aerosol generation above matched, specific pre-replicate baseline values for all test conditions. Nonparametric statistical techniques were used due to small sample sizes, with Bonferroni correction for multiple comparisons. Prism (v 8; GraphPad Software) was used to graph data. All values are reported as means with standard error.

## Results

### Mastoidectomy (No Barrier) With and Without an SS

**Figure 4.**
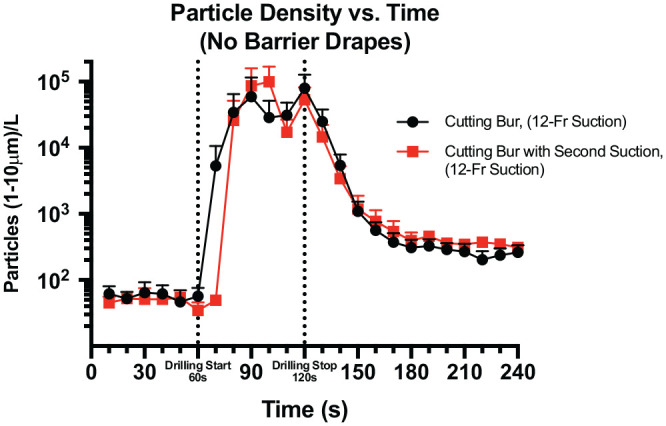
Mean ± SE particle density across time for mastoidectomy without a barrier in 2 conditions: cutting bur and cutting bur with a second suction.

The mean particle density across time is shown for mastoidectomy without a barrier drape in 2 drilling conditions: cutting bur and cutting bur with an SS (**Figure 4**). The mean particle (1-10 µm) density during 60 seconds of drilling—as detected 30 cm away from the surgical site in an open field without a barrier drape and with a cutting bur, with and without an SS—was 61,500 ± 19,200 and 42,500 ± 17,700 particles per liter, respectively.

The background level of particle detection was low prior to drilling in both conditions. The peak particle density occurred in a delayed fashion in both conditions, with maximum particle density noted at 30 seconds after drilling for the no-SS condition and at 40 seconds after drilling for the SS condition. No statistical difference was found between the conditions for particle density over a 60-second drilling period.

### Mastoidectomy With Barrier Drapes With and Without SS

**Figure 5.**
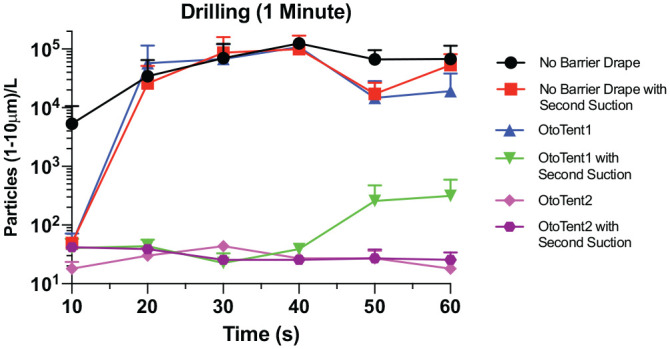
Comparison of particle density generated in mastoidectomy across time: without a barrier and with OtoTent1 and OtoTent2 and with and without a second suction. Values are presented as mean ± SE.

**Figure 6.**
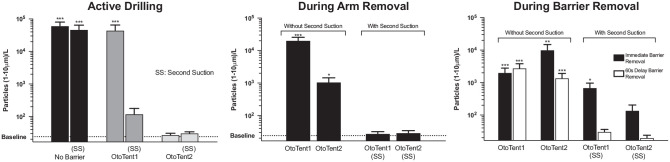
Particle density generated during 1 minute of drilling and following barrier removal, either immediately or after 1 minute had elapsed after drilling. Values are presented as mean ± SE. *P < .05. **P < .01. ***P < .001.

**Figure 5** presents the comparison of particle density generated in the mastoidectomy without a barrier drape and the 2 barrier strategies, OtoTent1 and OtoTent2, with and without the use of an SS. Three conditions—mastoidectomy without barrier drape (*P* < .001, *U* = 57), mastoidectomy without barrier drape but with an SS (*P* < .001, *U* = 95), and OtoTent1 without an SS (*P* < .001, *U* = 107)—showed high rates of particle generation during drilling as compared with background levels of particle density (n = 24 per condition, Mann-Whitney *U* test, Bonferroni correction for multiple comparisons). The remaining conditions (OtoTent1 with SS, OtoTent2 without SS, and OtoTent2 with SS) showed lower levels of particle generation during drilling, and the number of particles generated was not found to be statistically different from that in background levels for each of these 3 conditions (**Figure 6**).

### Effect of Arm Removal From Drape

During surgeon arm removal, OtoTent1 and OtoTent2 resulted in significant aerosol dispersion above background (*P* < .001, *U* = 0, and *P* < .05, *U* = 24.5; **Figure 6**), but when the SS was used, the levels were not significantly different from background.

### Effect of Delaying Barrier Removal

The effect of delaying barrier removal by 60 seconds following completion of drilling is shown in **Figure 6**. Delaying barrier removal with OtoTent1 without an SS still demonstrated significant aerosol dispersion when compared with background levels (*P* < .001, *U* = 0, n = 10, 12). Although delaying barrier removal with OtoTent2 without an SS marginally reduced aerosol generation as compared with immediate removal, significant aerosol was still generated as compared with background levels (*P* < .001, *U* = 2, n = 12, 12). However, delaying barrier removal with OtoTent1 with an SS or OtoTent2 with an SS mitigated aerosol dispersion to levels not significantly different from baseline.

## Discussion

This study demonstrates that mastoid drilling generates large quantities of 1- to 10-µm aerosolized particles, complementing existing research of larger particles generated during mastoidectomy.^6,10^ Concerns that COVID-19 may be spread through otologic and neurotologic surgery have arisen,^14^ as the fluid and mucosa of the middle ear and mastoid are contiguous with that of the upper respiratory tract, where the viral load is high.^2^ Other respiratory viruses—such as human coronavirus, rhinovirus, respiratory syncytial virus, influenza, parainfluenza, enterovirus, and adenovirus—have been identified in middle ear fluid samples from children with upper respiratory illnesses.^15,16^ Although we are unaware of studies showing SARS-CoV-2 in the middle ear, it is prudent to assume a potential risk of otologic transmission. While SARS-CoV-2 is primarily spread via droplet transmission,^17^ it can act as an opportunistic airborne infection, particularly in the setting of aerosolizing procedures.^9,18^ Typically, airborne aerosol particles are <5 µm, while droplet spread occurs through particles >5 µm.^13^

Mastoidectomy appears to generate far more aerosol dispersion than speech, cough, and sneeze, as well as intubation, intranasal cautery, and anterior skull base drilling, within the limits of comparison.^19,20^ There is a paucity of experimental data for small-particulate mastoidectomy aerosolization, and our data could not be compared with a prior study with a gravitational spectrometer,^7^ due to differences in mass-based rather than optical particle size quantification. Risks from aerosol-generating procedures may be stratified into a “high risk” category, which denotes increased risk based on (1) viral load at that site, (2) degree of aerosolization, and (3) exposure time.^21^ While viral load in the mastoid/middle ear is unknown for SARS-CoV-2, this study suggests that a high degree of aerosolization and prolonged exposure time may occur in otologic and neurotologic cases.

We investigated the use of 2 barrier strategies to mitigate aerosols produced during mastoidectomy. Both could be attached to any microscope and some exoscopes. OtoTent1 was created from a commercially available low-cost opaque drape, and the design is described in a prior study.^6^ Carron et al proposed implementation of 2 similar barrier drape concepts that used either a 1015 Steri-drape (3M) or a C-Armor drape (Tidi), and Hellier et al recommended that a second microscope drape be used to reduce droplet spray.^22,23^ These innovations suggest that otolaryngologists are interested in techniques that mitigate aerosol and droplet dispersion. Unfortunately, simple barrier drapes can be inconvenient to use, preventing easy passage of instruments and intermittently obscuring the surgical field.

Thus, we created a customized drape, OtoTent2, to address usability issues and potentially improve airborne aerosol containment. Designed with clear plastic, OtoTent2 has specialized ports for the surgeon’s arms and ports to accommodate easy transfer of instruments between the surgical scrub technician and the surgeon. OtoTent2 forms a semienclosed space over the surgical site, including a partial “floor” with a central hole to access the surgical site. OtoTent2 is not sealed around the surgical site and can be lifted off the field without dripping any pooled irrigation fluid. Irrigation runoff can be managed per the surgeon’s current preferred setup (ie, with a separate irrigation collection bag or with towels placed around the drilling site). OtoTent2 includes a rigid frame to keep the operating space unobstructed by drape material. Surgeons who trialed OtoTent2 in the laboratory reported that it was comfortable to use and did not obstruct the view of the surgical site.

OtoTent2 without SS successfully contained aerosol during 1-minute drilling trials such that the mean particle density was not significantly different from background levels. In OtoTent2, the “floor” and the use of arm and instrument ports likely accounted for improved aerosol containment, but the individual design elements were not evaluated separately to determine which features were effective. When OtoTent1 was used without SS, high aerosol levels were measured versus background, which may have been from aerosol escape from under the open edge of OtoTent1 and escape with small arm movements. Thus, while OtoTent1 may successfully mitigate large-droplet splatter,^6^ it does not appear to successfully decrease small-particle spread.

Placement of the SS within the drape is critical for decreasing particle dispersion, likely due to increased volume of air turnover within the drape. The volume of OtoTent1 and OtoTent2 barrier drapes were approximately 40 L and 37 L, respectively. The flow rate of the SS was 65 L/min such that volume within the drape could be exchanged during drilling. In contrast, the flow rate of the 12-Fr suction was 32 L/min.

Use of the SS within OtoTent1 reduced aerosol dispersion such that, on average, aerosol density was not significantly greater than baseline. There was, however, some variability in aerosol dispersion in the trials with OtoTent1 with an SS, which may be attributed to inconsistencies in OtoTent1 “seal” at the bottom edge of the open drape or around the arms, depending on positioning. Use of the SS and delayed removal of the drape both appear to be important for minimizing aerosol escape during surgeon arm removal and drape removal. Overall, simultaneous application of multiple strategies was important, including (1) use of the barrier drape, (2) increased air turnover via the SS, and (3) delayed drape removal.

Potential concerns with using a barrier drape include added time for setup, difficulty in passing instruments, concerns with the drape obstructing the view, particulate accumulating on the drape/lens, and interference with management of an unexpected adverse event (eg, injury to the sigmoid sinus). OtoTent1 and OtoTent2 each take about 1 minute to set up. OtoTent2 improves ease of instrument exchange with the use of ports. Subjectively, the scaffold on OtoTent2 provides adequate rigidity such that the drape does not obstruct the surgical view, while OtoTent1 can temporarily obstruct the surgical view. Particulate accumulation on the drape does not appear to interfere with surgery, and debris on the lens can be wiped clean as needed. In the case of an adverse event, such as hemorrhage, instruments may be passed through the ports; the microscope with the attached drape may be moved away to access the surgical site; or the drape may be removed in a matter of seconds.

Overall, surgeons and operating room staff will need to balance concerns with potential risks of inhaling biomaterials—which, at the time of this writing, includes the potential risk of contracting SARS-CoV-2—with the inconveniences from using a drape. As testing availability and accuracy improve for COVID-19, the immediate threat of contracting the virus is reduced. However, the COVID-19 era has already led to heightened awareness of biomaterial dispersion from aerosol-generating procedures,^6,10,19,20^ which may lead to long-term changes in practice patterns despite a lack of proven nosocomial infections.

The limitations of this study stem from the use of static methods for aerosol assessment, cadaveric models, and the natural variability in aerosol generation from high-speed drilling. This study measured optical particle size without the use an aerodynamic particle sizer or dynamic assessment techniques. Particulate density was measured at only 1 location in the surgical field, and particulates >10 µm in size were not assessed. Small droplets and bone dust particulate could not be distinguished. The presence of infectious pathogens, including virus or bacteria, in the aerosol was not assessed. Simulation in the laboratory setting did not completely replicate operating room settings, such as the rate of air exchange turnover, longer drilling times, drilling in living patients (with viable mucosa and mucous), and instrument exchange (ie, drill burs and suctions). Further research is needed to determine the optimal length of the rest period prior to drape removal. Despite the apparent success of the barrier strategies, PPE should not be reduced, as this study has not been replicated in a clinical setting.

## Conclusions

Mastoidectomy with a high-speed drill is a highly aerosolizing procedure with the potential to disperse particles <10 µm. Barrier drapes can be an effective way to mitigate aerosol dispersion, but this depends on the drape design. Use of OtoTent2 barrier drape was an effective strategy to mitigate dispersion of airborne aerosols during drilling. With the addition of an aerosol-scavenging SS, the average measured aerosol did not rise significantly above baseline with either drape, even during arm removal from under or inside the drape. The combination of an SS and a delayed drape removal after drilling should be employed to decrease particle dispersion during removal of either drape. These 3 strategies—barrier drape, SS, and delayed drape removal—may be used as an adjunct to appropriate PPE during the COVID-19 era.
